# Bayesian Piecewise Linear Mixed Models With a Random Change Point: An Application to BMI Rebound in Childhood

**DOI:** 10.1097/EDE.0000000000000723

**Published:** 2017-09-28

**Authors:** Samuel L. Brilleman, Laura D. Howe, Rory Wolfe, Kate Tilling

**Affiliations:** From the a Department of Epidemiology and Preventive Medicine, Monash University, Alfred Centre, Melbourne, VIC, Australia; b Victorian Centre for Biostatistics (ViCBiostat), Melbourne, VIC, Australia; c School of Social and Community Medicine, University of Bristol, Bristol, United Kingdom; and d MRC Integrative Epidemiology Unit at the University of Bristol, University of Bristol, Bristol, United Kingdom.

## Abstract

**Background:**

Body mass index (BMI) rebound refers to the beginning of the second rise in BMI during childhood. Accurate estimation of an individual’s timing of BMI rebound is important because it is associated with health outcomes in later life.

**Methods:**

We estimated BMI trajectories for 6545 children from the Avon Longitudinal Study of Parents and Children. We used a novel Bayesian two-phase piecewise linear mixed model where the “change point” was an individual-level random effect corresponding to the individual-specific timing of BMI rebound. The model’s individual-level random effects (intercept, prechange slope, postchange slope, change point) were multivariate normally distributed with an unstructured variance–covariance matrix, thereby, allowing for correlation between all random effects.

**Results:**

Average age at BMI rebound (mean change point) was 6.5 (95% credible interval: 6.4 to 6.6) years. The standard deviation of the individual-specific timing of BMI rebound (random effects) was 2.0 years for females and 1.6 years for males. Correlation between the prechange slope and change point was 0.57, suggesting that faster rates of decline in BMI prior to rebound were associated with rebound occurring at an earlier age. Simulations showed that estimates from the model were less biased than those from models, assuming a common change point for all individuals or a nonlinear trajectory based on fractional polynomials.

**Conclusions:**

Our model flexibly estimated the individual-specific timing of BMI rebound, while retaining parameters that are meaningful and easy to interpret. It is applicable in any situation where one wishes to estimate a change-point process which varies between individuals.

During early life, humans typically experience two periods of increasing body mass index (BMI) and one period of decline. The first period of increase is generally quite rapid and occurs during the first year of life. From around 1 year of age, BMI gradually declines for several years. “BMI rebound” refers to the time at which the child’s BMI stops decreasing and instead starts increasing for a second time, an increase that continues into adulthood.^1^ For most children, this will occur around 6 years of age; however, there is relatively large heterogeneity between individuals. It is important to be able to accurately identify the individual-specific timing of BMI rebound because it is associated with health outcomes in later life, including risk factors for chronic disease. For example, early BMI or adiposity rebound has been shown to be associated with an increased risk of subsequent obesity,^1–4^ type 2 diabetes,^5^ and potentially also cardiovascular disease.^6^

A statistical framework for estimation of the timing of BMI rebound is provided by piecewise linear mixed modeling. The standard two-phase piecewise linear mixed model is limited by the fact that the “change point,” defined as the time at which a change in slope occurs, is common across all individuals. A number of authors have, therefore, extended the model to treat the change point as a random effect parameter, thereby, allowing individuals to have their own change point.^7–12^ The use of a random change point has the advantage of increasing model flexibility and is, therefore, likely to improve model fit without major alteration of parameter interpretation. Such models provide useful insights when the person-specific timing of the change point is of intrinsic interest, for example, estimating the onset of cognitive decline in the elderly^7,12,13^ and disease progression in HIV patients.^9,10^

Piecewise linear mixed models with a random change point have predominantly been estimated using a Bayesian approach,^7–10,12^ although frequentist estimation techniques have also been proposed.^11^ Extensions to these models have been considered, for example, the use of smooth changes in slope around the random change point,^13^ multiple random change points,^14^ mixtures of linear and piecewise linear models,^15^ or the incorporation of a random change point model in the context of joint modeling of longitudinal and time-to-event data.^16–18^ However, a limitation of the random change point model when used in epidemiologic research has been a preference, presumably on pragmatic computational grounds rather than on any inherent substantive rationale, to not allow all individual-level random effects to be correlated; for example, assuming the random change point is independent of other individual-specific parameters in the model, such as the rate of growth.

In this article, we present a two-phase piecewise linear mixed model with a random change point, which we use to estimate longitudinal BMI trajectories for children aged between 1 and 15 years. The random change point in this model corresponds to the individual-specific timing of BMI rebound in childhood. We extend previous approaches by estimating an unstructured correlation matrix across the model’s four individual-level random effects (intercept, prechange slope, postchange slope, and change point), thereby, gaining additional insights. We estimate our model under a Bayesian framework using the statistical software Stan.^19^ In a simulation study, we compare our random change point model to an alternative model based on fractional polynomials, as well as simpler change point models that do not allow for between-individual variability in the timing of BMI rebound.

## METHODS

### The Avon Longitudinal Study of Parents and Children

The Avon Longitudinal Study of Parents and Children (ALSPAC) is a prospective birth cohort that enrolled expectant mothers in southwest England who were due to give birth between 1 April 1991 and 31 December 1992. A detailed description of the ALSPAC cohort, including the recruitment process, has been described elsewhere,^20^ and the ALSPAC website contains details of all the available data through a fully searchable data dictionary (http://www.bris.ac.uk/alspac/researchers/data-access/data-dictionary/). Ethical approval for this study was obtained from the ALSPAC Ethics and Law Committee and the Local Research Ethics Committees.

### Model Formulation

Let *y*_*ij*_ = *y*_*i*_(*t*_*ij*_) denote the observed BMI measurements taken for the 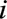
th child (*i* = 1,…, *N*) at some time points *t*_*ij*_ (*j* = 1, …, *n*_*i*_) measured in years. We define BMI as weight (in kilograms) divided by the square of height (in meters^2^). We model the observed BMI measurements using a piecewise linear mixed effects model of the form


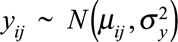



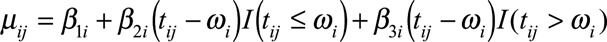
(1)

where 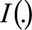 is the indicator function, 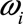 is the individual- specific change point, 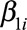 is the individual-specific intercept denoting the expected value of BMI at the change point, 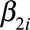 is the individual-specific linear slope before the change point (prechange slope), and 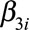 is the individual-specific linear slope after the change point (postchange slope). The individual-specific random parameters 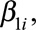 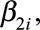 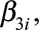 and 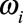 can be further specified as


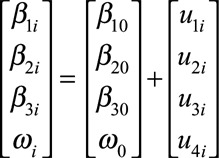
(2)

such that 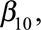 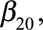 and 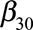 represent the fixed (population average) intercept, prechange slope, and postchange slope parameters, 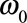 represents the fixed (population average) change point, and 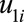 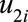 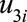 and 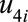 are the individual-level random effects (or deviations from the population average) associated with those parameters. We assume the vector of individual-level random effects 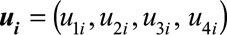 is multivariate normally distributed with mean zero and an unstructured variance–covariance matrix


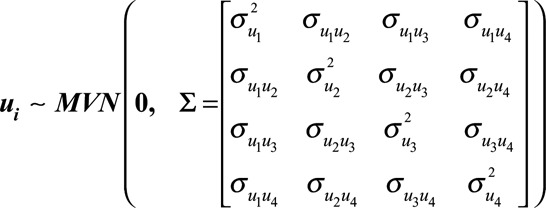
(3)

In other words, our model allows for correlation between the random intercept, prechange slope, postchange slope, and change point parameters. We denote the corresponding correlation matrix for the random effects as


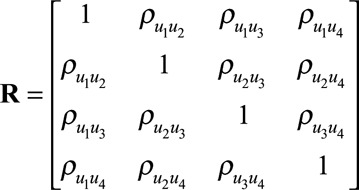


### Model Estimation

We adopt a Bayesian approach for estimating our model that we implement in the software Stan.^19^ Following the advice of Gelman, we use weakly informative prior distributions for the regression coefficients.^21^ We use the separation strategy to decompose the random effects variance–covariance matrix 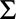 into a correlation matrix 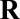 and separate standard deviation terms for each of the random effects (
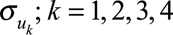
).^22^ This allows us to specify prior distributions separately for each of these components in a much more intuitive way. We use the “LKJ” correlation matrix distribution, implemented in Stan, as a prior distribution for the random effects correlation matrix.^23,24^ We used R Version 3.1.3 for preprocessing of data and for postprocessing and analysis of the MCMC samples.^25^ We interface with Stan from R using the RStan package.^19^ The eAppendix; http://links.lww.com/EDE/B247 (Sections 1 and 2) contains further details of the model implementation (for example, prior distributions and computation) and the code for fitting the random change point model; http://links.lww.com/EDE/B242; http://links.lww.com/EDE/B246.

We graphically present the estimated BMI trajectories in two ways. First, we plot the 95% credible interval (i.e., the 2.5th and 97.5th percentiles) of the posterior predictive distribution given by


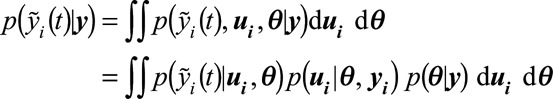
(4)

where 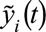 is a newly generated BMI measurement under the model (i.e., an in-sample prediction) for the 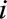
th child at time 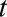, 
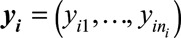
 denotes the vector of observed BMI measurements for the 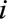
th child, 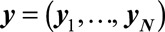 denotes the vector of observed measurements for all children, 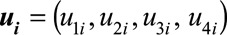 is the vector of random effects for the 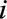
th child, and 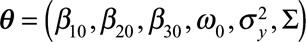 denotes the vector of all remaining unknown model parameters. Since the new data is assumed to be independent of the observed data given the model parameters, the 
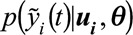
 term in equation (4) does not need to condition on 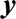. Further, by integrating over the random effects 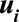 and the hyperparameters 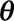, the posterior predictive distribution incorporates uncertainty associated with each of the parameters estimated under the model.

We also plot the expected BMI trajectory calculated using the posterior mean for each of the model parameters defined as 
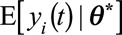
 for the 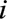
th child at time 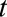, where 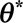 denotes the complete vector of posterior means for all parameters in the model, including random effects. This trajectory will exhibit the abrupt change in slope at the change point, which is characteristic of the piecewise linear mixed model, because the estimates are calculated using a unique realization of the model parameters. Conversely, predictions from the posterior predictive distribution are based on the joint posterior distribution for all model parameters (including the random change point) and, therefore, may exhibit apparent nonlinearity around the change point.

### Comparison With Alternative Models

In a simulation study, we compared the performance of our random change point model to simpler alternative change point models that have been commonly used. This includes a model that assumes a fixed (common) change point for all individuals or one that assumes the random change point is independent from the other individual-level random effects. When generating the data for our simulation study, we assumed that there is true underlying heterogeneity between individuals in terms of when BMI rebound occurs. The simulation study, therefore, allows us to quantify the bias that may be induced by not appropriately allowing for between-subject variability in the timing of the change point. In addition, we compared our random change point model to a complex alternative that allows for flexible BMI trajectories through the use of fractional polynomials. The models were compared using data generated according to two different processes: one based on our random change point model and the other based on the fractional polynomial model.

## RESULTS

A total of 14,701 children in the ALSPAC cohort were alive at 1 year of age. In our analysis, we include those children who had at least eight BMI measurements taken between ages 1 and 15 years and analyze data for females and males separately. Therefore, our analysis includes 3248 female and 3297 male children, with a total of 38,686 female and 39,367 male BMI measurements. The mean (maximum) number of BMI measurements per child was 11.9 (35) for females and 11.9 (34) for males. Variation in the observed BMI measurements generally increased with age, and the lowest overall mean BMI was observed between ages 5 and 7 years (Table 1). The Figure shows the observed BMI trajectories for 10 female children in the ALSPAC cohort.

**TABLE 1.**
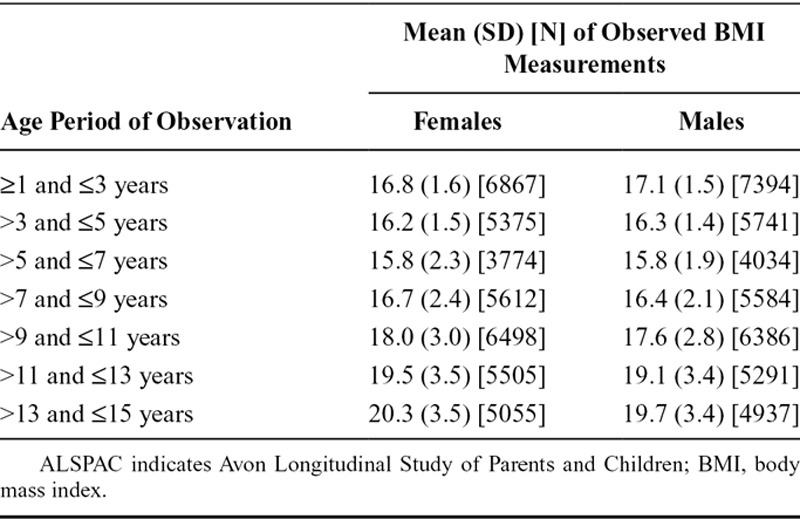
Mean, Standard Deviation, and Number of Observed BMI Measurements for Individuals in the ALSPAC Cohort, Stratified by Age Period of Observation and Gender

**FIGURE.**
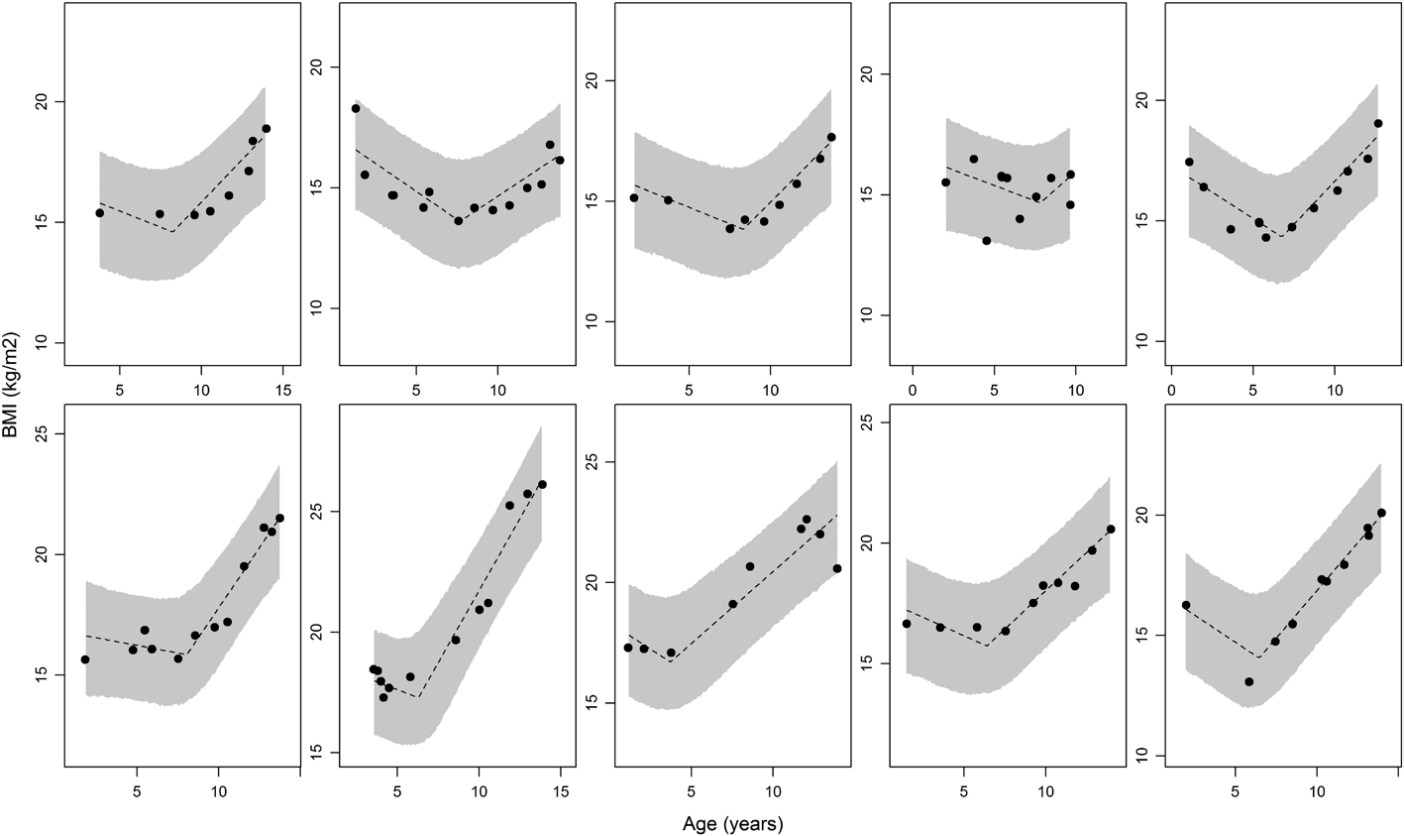
Observed BMI data and estimated BMI trajectories (under the random change point model) for 10 female children in the ALSPAC dataset. The dashed line represents the estimated BMI trajectory based on the posterior mean for each of the parameters in the model, while the shaded area represents the 95% credible interval associated with the posterior predictive distribution for that child.

We used the random change point model to estimate individual-specific changes in BMI between ages 1 and 15 years. Table 2 shows the estimated parameters from the fitted model, for females and males separately (95% credible intervals are shown in the table; however, these are omitted from the following text to aid readability). The estimated mean BMI when rebound occurs is 15.28 and 15.25 kg/m^2^ for females and males, respectively. The estimated mean rate of change in BMI prior to, and following, rebound is −0.36 and 0.75 kg/m^2^ per year for females, while the corresponding estimates for males are −0.43 and 0.63 kg/m^2^ per year. The estimated mean change point, which is the age at which BMI rebound is estimated to occur for the average individual, is 6.5 years for both females and males. There appears to be relatively large variability between individuals in terms of the age at which BMI rebound occurs, with the standard deviation for the random change point estimated at 2.0 years for females and 1.6 years for males. There was a moderately strong positive correlation (0.57 for both females and males) between the random effects for the prechange slope and the change point itself, suggesting children with a faster rate of decline in BMI prior to rebound are likely to experience rebound occurring at an earlier age.

**TABLE 2.**
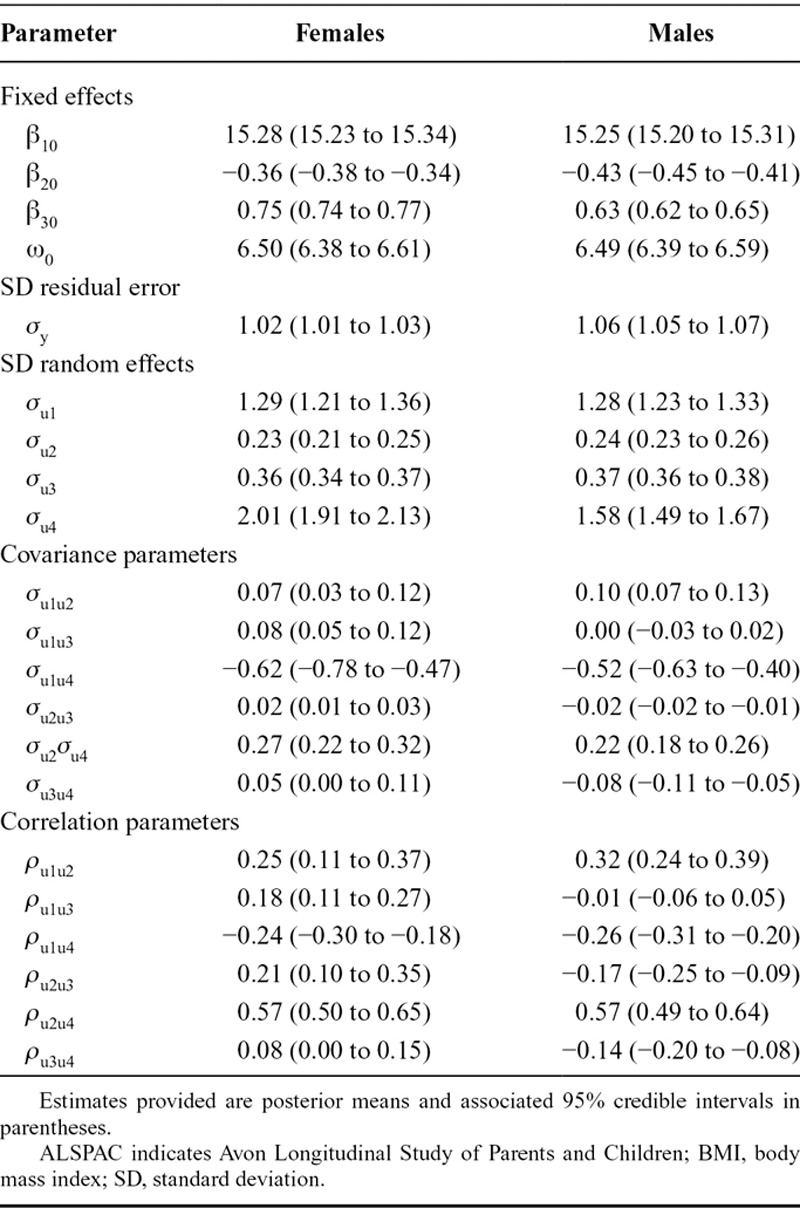
Parameter Estimates From the Piecewise Linear Mixed Model With a Random Change Point When Used to Model Changes in BMI Between Ages 1 and 15 Years for Male and Female Children in the ALSPAC Cohort

The Figure shows as dashed lines the estimated BMI trajectories for 10 female children calculated at the posterior mean for each of the model parameters, while the shaded area represents the 95% credible interval of the posterior predictive distribution. The model fits the observed data well, with the majority of data points fitting within the 95% credible limits for the posterior predictive distribution. The between-child variation in the random change points is evident from the plots. In the eAppendix; http://links.lww.com/EDE/B247 (Section 3), we have provided several plots of the standardized residuals from the fitted model. The residuals appeared to be normally distributed with no obvious patterns over time and constant variance, suggesting an adequate model fit across the entire age range.

In the eAppendix; http://links.lww.com/EDE/B247 (Section 4), we describe in detail the results from our simulation study. In brief, we found that a fractional polynomial model, when fitted to data generated under a random change point process, severely underestimated the mean timing of BMI rebound (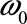 was estimated as the turning point of the fractional polynomial model and resulted in relative bias of approximately −20%). However, the estimate of the mean timing of BMI rebound obtained from the random change point model was much less biased, even when the true data-generating process was based on fractional polynomials (relative bias for 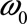 of approximately −2%).

When comparing several alternative change point models, we found that a model with a fixed (common) change point for all individuals resulted in the largest increases in bias, and this was relevant for both fixed and random effect parameters. Models with a random change point were much less biased, but we did find that as the true correlation between the random change point and the other individual-level random effects increased, there was increasing bias in the estimated parameters from a model which wrongly assumed that the change point was independent. While the covariance and correlation parameters for the random effects were most severely impacted, the fixed-effect regression coefficients were also affected.

## DISCUSSION

In this article, we have used a piecewise linear mixed model with a random change point to estimate BMI trajectories across childhood for 6545 children from the ALSPAC study. The timing of BMI rebound is a biologic characteristic known to vary between individuals. Through the use of a random change point, our model provided the flexibility required to estimate the individual-specific timing of BMI rebound for each child, while also providing an estimate of the mean timing of BMI rebound and the variability around that mean. The estimated parameters in our model, for example, the individual-specific (and average) intercepts, slopes, and change points, all remain easily interpretable. Alternative models that allow for flexible nonlinear trajectories (through the use of, say, polynomials, nonlinear splines, or other nonlinear functions, such as the SuperImposition by Translation And Rotation (SITAR) model^26^) may fit observed data better, but the interpretation of parameter estimates is often problematic and the generalizability of increasingly tailored models may be questioned.

Previous studies aimed at identifying the timing of BMI rebound have taken a variety of approaches. Relatively simple approaches such as the “visual inspection method” have been used, whereby the minimum of the BMI curve is assessed visually using the observed data but without any fitted model.^27,28^ When using the visual inspection method, the timing of the rebound is limited to those ages at which a BMI value is observed, and there will be varying amounts of measurement error depending on the number and timing of ages of measurement. Modeling approaches, on the other hand, allow the timing of the rebound to be estimated as having occurred between observation time points. Nonetheless, it has been suggested that the visual inspection method may more appropriately capture the physiological basis for BMI or adiposity rebound.^29^ This is because individuals who have a prolonged period of minimum BMI (a “plateau”) will have the timing of the rebound estimated at the end of the plateau under the usual criteria for the visual inspection method but estimated closer to the centre of the plateau (in other words at an earlier age) under most modeling approaches.

The most common modeling approach for estimating BMI rebound has been the use of polynomial functions for modeling changes in BMI over time.^4,29–31^ Wen et al. used linear mixed models with fractional polynomials to model BMI trajectories across childhood.^30^ They estimated BMI rebound using the individual-level turning point for the fitted polynomial function. One difficulty with the use of a fitted polynomial function, however, is that it does not directly provide easily interpretable slope estimates corresponding to the rates of change in BMI at various stages of childhood (although it would be possible to explicitly calculate the slope estimates for each individual at a specific set of discrete ages and summarize these values). An additional advantage of our modeling approach is that it allows us to succinctly quantify variation in the timing of BMI rebound. For example, under the assumption of normally distributed random effects, we estimated the standard deviation of the timing of BMI rebound as 2.0 years for females and 1.6 years for males. Further, in our simulation study (eAppendix; http://links.lww.com/EDE/B247, Section 4), we found that an alternative analysis model based on fractional polynomials only provided an unbiased estimate of the mean timing of BMI rebound when the true data generating process was also based on fractional polynomials. However, our random change point model provided relatively unbiased estimates of the mean timing of BMI rebound across two different data generating processes: one based on the random change point model and the other based on fractional polynomials.

The model used in this study was estimated using an unstructured variance–covariance matrix for the individual-level random effects. A simplistic alternative to estimating an unstructured variance–covariance matrix is to assume independence between some or all of the individual-level random effects by setting their respective pairwise correlations to zero. For example, Muniz Terrera et al.^12^ and Kiuchi et al.^9^ allow for a 3 × 3 unstructured covariance matrix for the random intercept and two random slopes but estimated the random change point independently. Muggeo et al.^11^ assumed a block diagonal covariance structure for the random effects, whereby they only allowed for two non-zero correlations. Other authors have used covariance structures with even greater restrictions.^7,8,13^ Although restricting some (or all) of the random effect correlation parameters to zero simplifies the model estimation, it does have the potential to bias results (see the results from our simulation study in the eAppendix; http://links.lww.com/EDE/B247). In addition, the estimation of all pairwise correlations between the individual-level random effects has the potential to provide benefits for interpretation, because in some settings, these correlation terms may be of direct interest.

Importantly, the most flexible random change point model we considered in the simulation study, which resulted in substantially less bias, required only four additional parameters to be estimated when compared with the model that assumed a common change point for all individuals. Nonetheless, estimating an unstructured covariance matrix can be computationally intensive when the random effects distribution is of a relatively high dimension. For example, in the case of a two-phase piecewise linear mixed model with a random intercept, two random slopes (prechange and postchange) and a random change point, the resulting unstructured variance–covariance matrix requires estimation of 10 parameters (four variances and six covariances). Furthermore, the variance of the residual error also needs to be estimated. In many epidemiologic studies, the requirements of estimating all of these parameters would be challenging, for example, due to a limited number of repeated measurements per individual. Convergence difficulties may also arise if the variances that need to be estimated are close to zero. In a Bayesian setting, the choice of prior distribution for the variance–covariance matrix can also pose difficulties. In this study, we used the Bayesian software Stan for fitting our model and discussed some of the computational benefits this provided. We are not aware of any paper that has discussed fitting this type of model using Stan or with the prior specification we used for the random effects distribution. In the eAppendix; http://links.lww.com/EDE/B247, we have provided the Stan code, as well as some simulated data, so that researchers can try fitting the model themselves (all software is freely available).

A further benefit of directly estimating parameters of key interest, such as the individual-specific change point, is that they can be used in turn to investigate their association with other exposures or outcomes. For example, one can investigate the association between the change point and later health outcomes, either through a joint modeling framework or a simpler two-stage process.^17^ Such extensions may be difficult when using other nonlinear modeling approaches such as polynomials or the SITAR model where parameters of key interest, such as the individual-specific timing of a change in growth, may not be directly estimated and may need to be derived. Importantly, if an estimate of the change point is to be used as the exposure in a subsequent model for later life outcomes, then the approach needs to incorporate the uncertainty in the estimated exposure. Ignoring this uncertainty may lead to overly precise and/or biased estimates of the effect of exposure on the later life outcome. Another related issue is that any measurement error in estimating age at BMI rebound as an exposure will bias estimates of associations with outcomes towards the null. Therefore, the more accurately BMI rebound can be estimated, the less biased the association with the outcome will be. The most appropriate approach is likely to be based on the use of a joint likelihood function for both the BMI trajectory model and the model for the later life outcome, as has been the main approach used for joint modeling of longitudinal and time-to-event data.^32,33^ However, some authors have found that in certain situations, a simpler (and less computationally intensive) two-stage approach may lead to very little bias, or in some cases no bias, even though it ignores the uncertainty in the estimated exposures.^34^

It is worth noting, however, that the ability to estimate a random change point model is likely to depend on the underlying statistical power for detecting changes in slope at the change point. In our application, we had no issues with model identifiability. However, in other settings where the change in slope is subtle or there is large between-individual variability in slopes before or after the change point, it may be difficult to identify the timing of the change point itself, which in turn could lead to model identifiability issues or problems achieving convergence. Such issues could be considered as part of future work.

## ACKNOWLEDGMENTS

We are extremely grateful to all the families who took part in the ALSPAC study, the midwives for their help in recruiting them, and the whole ALSPAC team, which includes interviewers, computer and laboratory technicians, clerical workers, research scientists, volunteers, managers, receptionists, and nurses.

## References

[1] Rolland-Cachera MF Deheeger M Bellisle F Adiposity rebound in children: a simple indicator for predicting obesity. Am J Clin Nutr. 1984;39:129 135. 6691287 10.1093/ajcn/39.1.129

[2] Ohlsson C Lorentzon M Norjavaara E Kindblom JM Age at adiposity rebound is associated with fat mass in young adult males-the GOOD study. PLoS One. 2012;7:e49404. 23166661 10.1371/journal.pone.0049404 PMC3498114

[3] Rolland-Cachera MF Deheeger M Maillot M Bellisle F Early adiposity rebound: causes and consequences for obesity in children and adults. Int J Obes (Lond). 2006;30(suppl 4):S11 S17. 17133230 10.1038/sj.ijo.0803514

[4] Whitaker RC Pepe MS Wright JA Seidel KD Dietz WH Early adiposity rebound and the risk of adult obesity. Pediatrics. 1998;101:E5. 10.1542/peds.101.3.e5 9481024

[5] Eriksson JG Forsén T Tuomilehto J Osmond C Barker DJ Early adiposity rebound in childhood and risk of Type 2 diabetes in adult life. Diabetologia. 2003;46:190 194. 12627317 10.1007/s00125-002-1012-5

[6] Singhal A Cole TJ Fewtrell M Promotion of faster weight gain in infants born small for gestational age: is there an adverse effect on later blood pressure? Circulation. 2007;115:213 220. 17179023 10.1161/CIRCULATIONAHA.106.617811

[7] Dominicus A Ripatti S Pedersen NL Palmgren J A random change point model for assessing variability in repeated measures of cognitive function. Stat Med. 2008;27:5786 5798. 18680123 10.1002/sim.3380 PMC4761443

[8] Hall CB Ying J Kuo L Lipton RB Bayesian and profile likelihood change point methods for modeling cognitive function over time. Comput Stat Data Anal. 2003;42(1–2):91 109.

[9] Kiuchi AS Hartigan JA Holford TR Rubinstein P Stevens CE Change points in the series of T4 counts prior to AIDS. Biometrics. 1995;51:236 248. 7766779

[10] Lange N Carlin BP Gelfand AE Hierarchical Bayes models for the progression of HIV-infection using longitudinal Cd4 T-cell numbers. J Am Stat Assoc. 1992;87(419):615 626.

[11] Muggeo VMR Atkins DC Gallop RJ Dimidjian S Segmented mixed models with random changepoints: a maximum likelihood approach with application to treatment for depression study. Stat Model 2014;14(4):293 313.

[12] Muniz Terrera G van den Hout A Matthews FE Random change point models: investigating cognitive decline in the presence of missing data. J Appl Stat 2011;38(4):705 716.

[13] van den Hout A Muniz-Terrera G Matthews FE Smooth random change point models. Stat Med. 2011;30:599 610. 21337356 10.1002/sim.4127

[14] Huang Y Dagne GA Zhou S Wang Z Piecewise mixed-effects models with skew distributions for evaluating viral load changes: a Bayesian approach. Stat Methods Med Res. 2015;24:730 746. 22045781 10.1177/0962280211426184

[15] Moss A Juarez-Colunga E Nathoo F Wagner B Sagel S A comparison of change point models with application to longitudinal lung function measurements in children with cystic fibrosis. Stat Med. 2016;35:2058 2073. 27118629 10.1002/sim.6845

[16] Ghosh P Vaida F Random changepoint modelling of HIV immunologic responses. Stat Med. 2007;26:2074 2087. 16969894 10.1002/sim.2671

[17] Jacqmin-Gadda H Commenges D Dartigues JF Random change point model for joint modeling of cognitive decline and dementia. Biometrics. 2006;62:254 260. 16542253 10.1111/j.1541-0420.2005.00443.x PMC2233714

[18] de Dieu Tapsoba J Lee SM Wang CY Joint modeling of survival time and longitudinal data with subject-specific changepoints in the covariates. Stat Med. 2011;30:232 249. 21213341 10.1002/sim.4107 PMC3059268

[19] Stan Development Team. Stan: A C++ Library for Probability and Sampling, Version 2.6.0. 2014.

[20] Boyd A Golding J Macleod J Cohort Profile: the ‘children of the 90s’–the index offspring of the Avon Longitudinal Study of Parents and Children. Int J Epidemiol. 2013;42:111 127. 22507743 10.1093/ije/dys064 PMC3600618

[21] Gelman A Prior distributions for variance parameters in hierarchical models. Bayesian Anal. 2006;1(3):515 533.

[22] Barnard J McCulloch R Meng XL Modeling covariance matrices in terms of standard deviations and correlations, with application to shrinkage. Stat Sin 2000;10(4):1281 1311.

[23] Lewandowski D Kurowicka D Joe H Generating random correlation matrices based on vines and extended onion method. J Multivar Anal 2009;100(9):1989 2001.

[24] Stan Development Team. Stan Modelling Language User’s Guide and Reference Manual, Version 2.6.2. 2015.

[25] R Core Team. R: A Language and Environment for Statistical Computing. 2015 Vienna, Austria: R Foundation for Statistical Computing.

[26] Cole TJ Donaldson MD Ben-Shlomo Y SITAR–a useful instrument for growth curve analysis. Int J Epidemiol. 2010;39:1558 1566. 20647267 10.1093/ije/dyq115 PMC2992626

[27] Dorosty AR Emmett PM Cowin Sd Reilly JJ Factors associated with early adiposity rebound. ALSPAC Study Team. Pediatrics. 2000;105:1115 1118. 10790472 10.1542/peds.105.5.1115

[28] Rolland-Cachera MF Deheeger M Guilloud-Bataille M Avons P Patois E Sempé M Tracking the development of adiposity from one month of age to adulthood. Ann Hum Biol. 1987;14:219 229. 3662424 10.1080/03014468700008991

[29] Kroke A Hahn S Buyken AE Liese AD A comparative evaluation of two different approaches to estimating age at adiposity rebound. Int J Obes (Lond). 2006;30:261 266. 16231016 10.1038/sj.ijo.0803143

[30] Wen X Kleinman K Gillman MW Rifas-Shiman SL Taveras EM Childhood body mass index trajectories: modeling, characterizing, pairwise correlations and socio-demographic predictors of trajectory characteristics. BMC Med Res Methodol. 2012;12:38. 22458308 10.1186/1471-2288-12-38 PMC3375197

[31] Williams S Davie G Lam F Predicting BMI in young adults from childhood data using two approaches to modelling adiposity rebound. Int J Obes Relat Metab Disord. 1999;23:348 354. 10340811 10.1038/sj.ijo.0800824

[32] Rizopoulos D Joint Models for Longitudinal and Time-to-Event Data: With Applications in R. 2012 Chapman & Hall/CRC: Biostatistics Series CRC Press.

[33] Tsiatis AA Davidian M Joint modeling of longitudinal and time-to-event data: An overview. Stat Sinica 2004;14(3):809 834.

[34] Sayers A Heron J Smith A Macdonald-Wallis C Gilthorpe M Steele F Tilling K Joint modelling compared with two stage methods for analysing longitudinal data and prospective outcomes: a simulation study of childhood growth and BP. Stat Methods Med Res 2014;26:437 452. 10.1177/0962280214548822 PMC5476230 25213115

